# Knowledge assessment tools in atopic dermatitis patient education: a scoping review

**DOI:** 10.1186/s13223-025-00970-7

**Published:** 2025-05-24

**Authors:** Jasmin Khela, Bethany Wilken, Yuka Asai

**Affiliations:** 1 Translational Institute of Medicine, Translational Medicine Graduate Program, Queen’s University, Kingston, ON Canada; 2 Translational Institute of Medicine, Department of Medicine, Queen’s University, Kingston, ON Canada; 3 Division of Dermatology, Department of Medicine, Queen’s University, Kingston, ON Canada

**Keywords:** Atopic dermatitis, Eczema, Patient education, Health education, Therapeutic education, Knowledge assessment, Knowledge outcome measures

## Abstract

**Background:**

Atopic dermatitis (AD) is a chronic and inflammatory skin disease which requires continuous self-management by patients and caregivers. Patient education in AD can improve the self-management practices, treatment adherence rates, and clinical outcomes of patients. Patient-reported outcome measures and objective clinical outcome measures have been used to assess the effectiveness of AD patient education interventions, however they have limited use in assessing learning outcomes, such as knowledge. The literature on knowledge outcome measures for AD patient education interventions has not been examined to date.

**Main:**

We performed a scoping review of the literature on knowledge assessment tools for AD patient education interventions following the PRISMA-ScR framework. Search databases included MEDLINE, Embase, CINAHL, Education Source, Web of Science, Grey Matters, Clinical Trials.gov, and the International Clinical Trials Registry Platform (ICTRP). Of the 3914 articles identified from the search strategy, 20 studies were eligible for data extraction and summarized by narrative synthesis. Most studies were randomised controlled trials originating in the United States, Europe, and Asia, and published in the years of 2003–2023. Researchers commonly evaluated caregivers’ knowledge of AD and included assessments of clinical outcome measures. Similar methods were employed for assessing subjective knowledge across studies. Likewise, studies measuring AD patient/caregiver objective knowledge used comparable methods. Multiple-choice and true/false question formats were used in objective knowledge assessments, and Likert-type scales were common for evaluating subjective knowledge. Objective knowledge assessments consisted of more questions than subjective knowledge outcome measures. Content assessed in knowledge outcome measures was relatively consistent across studies. Delivery of subjective and objective AD knowledge assessments was by telephone, in clinic, and/or online. In pre- and post-test study designs, identical knowledge outcome measures were administered.

**Conclusion:**

This scoping review highlights the diverse components of knowledge assessment tools for AD patient education interventions. Further studies on developing and validating high-quality AD knowledge outcome measures are needed for assessing the true effects of patient education interventions on improving patient/caregiver knowledge.

## Introduction

Atopic dermatitis (AD), commonly referred to as eczema, is a chronic, relapsing–remitting, and inflammatory skin disorder with a lifetime prevalence of approximately 10–20% among the Canadian population [1–3]. AD is characterized by severe pruritus, dry skin, scaling, erythema, serous oozing, and blister formation [2, 4]. AD has a significant burden on the quality of life and psychological well-being of patients and caregivers [5]. AD is commonly diagnosed during childhood, although patients may also be affected during their adult years [2]. The management of AD is complex given that the disease often presents with alternating periods of quiescence and flares, which requires treatments tailored to both acute exacerbations and long-term maintenance [6, 7]. Many patients with AD can be successfully treated with the application of topical therapies, including emollients, corticosteroids, and non-steroid prescriptions, either as monotherapy or in combination with other treatments [8]. Continuous self-management of AD by patients and caregivers is essential, and includes applying the appropriate therapies at the right times to specific areas(s) of the skin, as well as avoiding triggering factors [9, 10]. Given the absence of a cure for AD and the complexity of treatment due to long-term therapy with multiple medications, frequent dosing schedules, and the cumbersome application of topical therapies, AD patients often experience frustration and despair and discontinue their course of treatment and/or reduce the frequency of topical therapy application to simplify treatment regimens [11]. Lack of understanding of the natural disease course in AD and its management can also contribute to poor treatment adherence and worsening AD symptoms, as patients may experience confusion about how to apply topical therapies correctly and escalate treatment when needed [5, 6, 11]. This may lead patients to believe that orthodox treatment strategies have failed and increase their willingness to try adjunct treatment options lacking in evidence for the management of AD [11, 12].

Patient education in AD can improve the self-management practices, treatment adherence rates, and clinical outcomes of patients [5, 13]. Traditionally, patient education in AD includes programs, workshops, and practical training addressing key aspects of disease management including topical corticosteroid application, choice of emollient, skin care and bathing practices [5]. Additionally, AD patient education facilitates a continuous process of patient-centered medical care, where there is concordance between the patient and care provider to construct an optimal treatment and disease management plan [9, 13]. Various studies have devised and evaluated novel patient education tools to improve outcomes in AD, and educational structures termed ‘eczema centres’ or ‘atopic schools’ have been developed by numerous hospital teams across the world [5, 14].

Patient-reported outcome measures (PROMs; subjective measures) have garnered interest in AD clinical trials and routine practice for use in tandem with objective clinical outcome measures, with research organizations in dermatology, such as the Harmonising Outcome Measures for Eczema (HOME), advancing the development of dermatology-specific measures for these purposes [15, 16]. A consensus-based core outcome set has been established for use in AD clinical trials to assess 1) signs/severity (Eczema Area and Severity Index (EASI) and SCORing Atopic Dermatitis (SCORAD); objective clinical outcome measures), 2) symptoms (Patient-Oriented Eczema Measure (POEM); PROM), 3) quality of life (Dermatology Life Quality Index (DLQI); PROM), and 4) eczema control (Atopic Dermatitis Control Tool (ADCT) and Recap of Atopic Dermatitis (RECAP); PROMs) [17, 18]. Many of these core outcomes have been implemented to assess the effectiveness of AD patient education interventions [19–23]. However, the core outcome set has limited use for assessing the effectiveness of AD patient education on learning outcomes, such as knowledge [14, 17].

Assessing knowledge as an outcome measure of AD patient education is an emerging consideration. Knowledge outcome measures can be used to directly assess the effectiveness of patient education interventions on learning outcomes, as they can identify the knowledge needs of patients, and the level of knowledge received during formal instruction [24]. Knowledge also forms the basis of patients’ cognitive judgement and their ability to make informed decisions about how to independently and effectively self-manage their disease [24, 25]. Acquiring adequate knowledge about AD and its management from formal instruction/patient education can enhance patients’ abilities to engage in self-care practices and self-observation to promptly recognize AD symptoms and flares and choose a suitable coping strategy (ex. moisturization and/or emollient use, application of topical therapies, use of systemic therapies, etc.) to improve their chronic disease outcomes [25, 26].

Wilken et al. [27] previously reviewed the characteristics of AD patient education interventions including the outcome measurements used, and identified measures that showed improvements among AD patients/caregivers after education, such as increased knowledge. Assessment of patient education in AD is an essential component of the educational process [14]. The objective clinical outcome measures and PROMs used after patient education have been detailed thoroughly in the literature. However, the methods employed by studies to evaluate knowledge after AD patient education interventions (i.e., how knowledge outcome measures have been designed and implemented for use in AD patient education interventions) have not been previously described. The forms of knowledge which are commonly used as outcome measures include an individual’s subjective/self-perceived knowledge (i.e., what one thinks they know) and objective knowledge (i.e., what one actually knows). Given that knowledge can be evaluated in various ways and knowledge outcome measures can provide indications for different learning outcomes, it is important to determine how knowledge assessment tools can be efficiently implemented for evaluating the effectiveness of patient education interventions. Scoping reviews are a form of knowledge synthesis that encompass broader explorations of a given topic by synthesizing an existing and/or emerging body of literature [28, 29]. This type of review employs a systematic or iterative approach similar to systematic reviews, but is used to map the extent, range, and nature of the literature on a subject, as well as determine possible gaps in the body of literature [29]. Scoping reviews are often employed to examine emerging evidence when it is still unclear what other more specific research questions can be developed and adequately addressed by a systematic review [29]. Since, the characteristics of knowledge assessment tools for AD patient education interventions have not been rigorously evaluated to date, a scoping review was conducted to systematically map and examine the extent and nature of evidence for knowledge assessment tools in AD patient education, as well as to identify any existing knowledge gaps and areas for further research [30]. The scoping review aimed to address the following question: *What is known from the literature about knowledge assessment tools in atopic dermatitis patient education?*

## Methods

Our protocol was drafted in accordance with the guidelines reported in the PRISMA extension for scoping reviews (PRISMA-ScR) [30].

### Eligibility criteria

Inclusion Criteria: Publications were required to assess knowledge as an outcome measure of receiving an AD educational intervention and composed in the English language. Studies which employed knowledge assessment tools for adult AD patients and parents/caregivers of pediatric AD patients were only included in this review. Articles which assessed AD patients’ knowledge after education as a primary or secondary outcome measure were incorporated.

Exclusion Criteria: Conference and poster abstracts without an associated full-text article were excluded from this review. Studies that did not evaluate patients’ knowledge as an outcome measure of receiving an AD educational intervention were excluded. Publications which evaluated knowledge as an outcome measure of educational interventions in contact dermatitis, allergic rhinitis, food allergy, as well as other related skin diseases were not included in this review.

### Information sources and search

To identify relevant sources for this review, the following databases were searched for publications and grey literature with no limits applied on publication date until January 11, 2024: Ovid MEDLINE, Embase, CINAHL, Education Source, and Web of Science. Clinical trials registered with ClinicalTrials.gov and the International Clinical Trials Registry Platform (ICTRP) were included in the search. The search strategies were drafted independently (J.K.) and further refined through collaboration with a librarian from Bracken Health Sciences Library. Similar keyword and search strategies were applied across all databases used. The search strategy for Ovid MEDLINE: (Dermatitis, Atopic/OR atopic dermatitis.mp OR atopic eczema.mp) AND ((health education/OR consumer health information/OR health literacy/OR patient education as topic/) OR health education.mp OR consumer health information.mp OR health literacy.mp OR (patient* AND education*) OR therapeutic education.mp OR action plan.mp). Search results were exported in Covidence for removal of duplicates and for publication screening. The search was supplemented by performing a snowball search.

### Selection of sources of evidence

Two reviewers (J.K. and B.W.) independently screened all titles and abstracts from Ovid MEDLINE, Embase, CINAHL, Education Source, and Web of Science, and searched Grey Matters, Clinical Trials.gov and the ICTRP. J.K. and B.W. independently examined full-texts of all publications identified by the searches for potentially relevant publications. Disagreements of study selection were resolved by a third reviewer (Y.A.), as necessary.

### Data charting and items

Twenty studies met criteria for inclusion in this review (J.K.). Characteristics of selected sources of evidence including the country of origin, study type, number of study participants, age and gender of participants, targeted population of the knowledge assessment, and clinical scores measured were charted in Table 1. Data on the content/topics evaluated in knowledge assessment tools, type of knowledge assessed, format of questions, number of questions, format of pre-education questionnaires in relation to post-education questionnaires, delivery method, scoring methods, completion time, as well as the questions asked in knowledge assessment tools (where available) were summarized in Table 2.

**Table 1:** Characteristics of studies included in the scoping review

Reference	Year	Country of Origin	Study Type	Number of Study Participants (Intervention/Control#)	Age of Participants (Mean)	Gender of Participants (Females/Males)	Knowledge Assessment Population	Clinical Scores Assessed
Andrade et al	2023	United States	Prospective	N/A	18 years or older	47/8	Patients and caregivers	N/A
Armstrong et al	2011	United States	RCT	40/40	50 years in intervention group; 46 years in control group	17/23 in intervention group; 19/21 in control group	Patients	POEM
Breuer et al	2014	Germany	RCT	274/244	Children—3 months to 7 years	N/A	Caregivers	SCORAD
Brown et al	2018	United States	RCT	11/26	Children—6 years in intervention group and 3 years in control group	5/6 in intervention group; 19/7 in control group	Caregivers	CDLQI, IDQOL
Chen et al	2023	United States	RCT	22/26	Children—3 years; caregivers—31 years in intervention group and 30 years in control group	13/9 in intervention group; 10/16 in control group	Caregivers	SCORAD
Cheong et al	2018	Singapore	Prospective	N/A	21–80 years of age	27/12	Caregivers	N/A
Cork et al	2003	United Kingdom	Prospective	N/A	Children—4 years	24/27	Caregivers	SASSAD
Dufresne et al	2020	France	Prospective	N/A	Children—6–12 years; adolescents—12–16 years	22/16	Patients	SCORAD
Gilliam et al	2016	United States	RCT	41/47	Children—4 years	21/20 in intervention group; 20/27 in control group	Caregivers	N/A
Jackson et al	2013	United Kingdom	Prospective	N/A	N/A	N/A	Caregivers	N/A
Jang et al	2015	South Korea	Prospective	N/A	Children—3–10 years	N/A	Caregivers	N/A
Johnson et al	2022	United States	Prospective	N/A	Children—8 months to 4 years	4/8	Caregivers	POEM
Joseph et al	2022	United States	RCT sub-study	24/24	Children—3 years; caregivers 29 years	12/12 in intervention group; 11/13 in control group	Caregivers	SCORAD
Liang et al	2018	China	RCT	291/249	Children—6 years	133/158 in intervention group; 114/135 in control group	Caregivers	SCORAD
								CDLQI, IDQOL
Rea et al	2018	United States	RCT	111/100	Children-1 month to 16 years	53/58 in intervention group; 43/57 in control group	Caregivers	POEM, IDQOL, CDLQI
Ryu and Lee	2014	South Korea	Prospective	32/66	Children—9 years; caregivers—40 years	21/11 in intervention group; 43/23 in control group	Caregivers	SCORAD, CDLQI
Shi et al	2013	United States	RCT	18/19	N/A	9/9 in intervention group; 8/11 in control group	Patients and caregivers	N/A
Shin et al	2014	South Korea	RCT	58/90	43 years	49/9 in intervention group, 78/12 in control group	Patients and caregivers	N/A
Singer et al	2018	United States	RCT	14/16	Children—1 year	5/9 in intervention group; 12/4 in control	Caregivers	EASI
Son and Lim	2014	South Korea	Quasi-experimental	20/20	Children—up until 3 years; caregivers—30–40 years	13/7 in intervention group; 12/8 in control group	Caregivers	POEM, IDQOL

**Table 2:** Characteristics of AD Knowledge Assessment Tools for Patient Education Interventions

Reference	Content/topics assessed	Type of knowledge assessed	Format of questions	Number of questions	Pre- and post-education questionnaires	Delivery	Scoring	Timing	Sample questions
Andrade et al. [31]	Cause of AD, common triggers of AD, pharmacological and non-pharmacological treatments for AD, dietary considerations, stress reduction/psychological impacts of AD	Objective	Dichotomous (true/false)	37	Identical pre- and post-education questionnaires	Online	Number-right	N/A	**1.** The more people scratch, the more it can lead to itching; **2.** Stress can make itching worse; **3**. Using oral steroids regularly for a long time is a great way to treat eczema; **4.** Using oral steroids regularly for a long time is the best way to treat severe itch in eczema; **5**. Oral and topical medications targeting skin inflammation is the best way to treat severe itch in eczema; **6.** Eczema affects the barrier provided by the skin; **7.** Eczema is mainly caused by an allergy to foods; **8.** Eczema can be treated with steroids taken by mouth or put on the skin; **9.** Dust and dust mites are common triggers for individuals living with eczema; **10.** Sweat can be a trigger for eczema attacks; **11.** Baths longer than 10 min provide a moisturizing effect on the skin; **12.** Detergents and soaps with fragrances can help with preventing eczema attacks; **13.** Wet wrap therapy can be done with moisturizers; **14.** Bleach and oatmeal baths are not helpful in treating eczema; **15.** We should moisturize after bathing or contacting water; **16.** Bar soaps are recommended versus liquid soaps; **17.** Topical antihistamines work great for itch in atopic dermatitis; **18.** When applying the treatments on your skin, one unit of topical corticosteroids is approximately the size of the tip of your index finger; **19.** When applying the treatments on your skin, the hand requires approximately 1 fingertip unit of corticosteroid for eczema attacks; **20.** When applying the treatments on your skin, the back and front of your trunk require 6–7 fingertip units each of corticosteroid for eczema attacks; **21.** Overall, biologic medications work by deceasing the inflammation in our bodies; **22.** All children with atopic dermatitis have food allergies; **23.** Children should eliminate foods considered ‘common allergens’ (eggs, wheat, soy) from their diets; **24.** A balanced diet involving vegetables, whole grains, healthy fats, and fish is the best diet for individuals with atopic dermatitis; **25.** Meditation and yoga are not helpful for symptoms of atopic dermatitis; **26.** Massage therapy for children can reduce symptoms of eczema attack and itch long-term; **27.** Wet wrap therapy can be used for periods longer than 2 weeks at a time; **28.** Wet wrap therapy includes applying the topical treatment to affected skin, covering skin with a wet layer, and covering the wet layer with dry fabric; **29.** Psychological treatments can be helpful for dealing with itch; **30.** Supplements, such as fish oil and vitamin D, have not been shown to improve symptoms of atopic dermatitis; **31.** Hives, itchiness, or swelling are signs of a food allergy; **32.** Anaphylaxis is a life-threatening allergic reaction that may present with wheezing, swelling of tongue or throat; **33.** Skin or blood allergy testing is the best way to determine active food allergy; **34.** Wool, polyester, and nylon are clothing products that do not irritate the skin; **35.** Pure cotton and silk are clothing products that do not irritate the skin; **36.** Steroids work by reducing an overactive immune system and decreasing swelling; **37.** Topical steroids should be avoided in areas with thinner skin, such as the face and skin folds
Armstrong et al. [5]	Clinical manifestations of AD, contributing environmental factors, bathing and hand-washing techniques, moisturizer vehicles, and common treatment modalities	N/A	N/A	14	Identical pre- and post-education questionnaires	N/A	N/A	N/A	N/A
Breuer et al. [20]	Medical, psychological and nutritional issues	Objective	Dichotomous (right/wrong)	49	Identical pre- and post-education questionnaires	N/A	Number-right	N/A	N/A
Brown et al. [7]	N/A	Subjective	Likert scale	N/A	Identical pre-and post education questionnaires	In clinic for pre-education questionnaire and by telephone for follow-up questionnaire	5-point scale	N/A	N/A
Chen et al. [32]	N/A	N/A	N/A	N/A	Identical pre- and post-education questionnaires	N/A	N/A	N/A	N/A
Cheong et al. [6]	Causes of eczema, trigger factors of eczema, definition of eczema flare, cure versus control of eczema, appropriate skincare, appropriate use of topical preparations, side effect of steroids	Objective	Dichotomous	24	Identical pre-and post-education questionnaires	In clinic for pre-education questionnaire and by telephone for follow-up questionnaire	Negative	N/A	**1.** One fingertip of medicated ointment/cream can cover one palm-sized area; **2.** Topical antibiotics should be applied whenever there is an eczema flare; **3.** Consult a doctor if there is no improvement after 1 week of continuous proper application of prescribed medicated cream; **4.** The exact cause of eczema is unknown; **5.** Eczema is commonly caused by food allergy
Cork et al. [13]	N/A	N/A	N/A	N/A	Identical pre- and post-education questionnaires	In clinic on paper	N/A	N/A	N/A
Dufresne et al. [33]	Definition, recognizing skin lesions, compounding environmental factors, understanding skin structures, environment and sportive activities	Objective	Multiple-choice and Dichotomous (true/false)	N/A	Identical pre- and post-education questionnaires	N/A	N/A	N/A	N/A
Gilliam et al. [34]	Skin care, treatment, prevention	Subjective	Likert scale	6	Identical post-education and follow-up questionnaires	In clinic for pre-education questionnaire and by telephone for follow-up questionnaire	5-point scale	N/A	**1.** I am sure which medicines or creams to use on my child’s body and which creams to use on my child’s face (0 = always, 1 = usually, 2 = sometimes, 3 = rarely, 4 = never); **2.** I know the names of the medicines or creams I am supposed to use for my child’s skin (0 = always, 1 = usually, 2 = sometimes, 3 = rarely, 4 = never); **3.** I feel certain about what to do when my child’s skin is getting worse (0 = always, 1 = usually, 2 = sometimes, 3 = rarely, 4 = never); **4.** I feel confident about deciding when my child needs urgent care for his/her skin condition (0 = always, 1 = usually, 2 = sometimes, 3 = rarely, 4 = never); **5.** I am aware that there are things I can do to help *prevent* my child’s skin condition from getting worse (0 = always, 1 = usually, 2 = sometimes, 3 = rarely, 4 = never); **6.** The teaching the doctor did during our clinic visit helped me to understand how to take care of my child’s skin condition (0 = always, 1 = usually, 2 = sometimes, 3 = rarely, 4 = never)
Jackson et al. [35]	Knowledge and attitudes, understanding of triggers, routines, changes in parents’ perceptions of steroid creams	Subjective	Focus group interview	N/A	Post-education interview only	In clinic	N/A	N/A	N/A
Jang et al. [43]	Understanding of AD	Subjective	Likert scale	1	Post-education questionnaire only	Telephone	5-point scale	N/A	**1.** Do you think your understanding of the disease is better? (Strongly agree, Agree, Neutral, Disagree, Strongly disagree)
Johnson et al. [44]	Knowledge and confidence of AD	Subjective	Likert scale	2	Post-education questionnaire only	Telephone	5-point scale	N/A	**1.** My knowledge and confidence regarding my child’s atopic dermatitis has improved after my initial atopic dermatitis visit. (1 = Strongly disagree, 5 = Strongly agree); **2.** My knowledge and confidence regarding my child’s atopic dermatitis has improved after the addition of the one-on-one nurse-led education and individualized eczema action plan. (1 = Strongly disagree, 5 = Strongly agree)
Joseph et al. [36]	Treatment, prevention, skin infection, wet wrap therapy, bleach baths	Subjective	Likert scale	5	Identical pre- and post-education questionnaires	In clinic	5-point scale	N/A	**1.** I know when my child’s eczema gets worse.; **2.** If the medicine the doctor gave me for my child does not work immediately, I stop using it.; **3.** I know when my child’s eczema has an infection; **4.** I know how to use bleach baths if the doctor tells me that my child should use them; **5.** I know how to apply wet wraps when the doctor tells me that we should use them
Liang et al. [37]	Use, functions, and ingredients of emollients	Subjective	Likert scale	9	Identical pre- and post-education questionnaires	N/A	4-point scale	N/A	**1.** Do you use emollients for your child’s skin care? (Very frequently, Frequently, Occasionally, Never); **2.** Do you think an emollient can relieve skin dryness? (Significantly, Moderately, Mildly, None); **3.** Do you think an emollient can improve skin pruritus? (Significantly, Moderately, Mildly, None); **4.** How frequently do you think emollients should be used? (Everyday, Every 2–3 days, 1 time per week, >1 time per week); **5.** How long do you think an emollient should be used? (1–3 months, About 6 months, About 1 year, >1 year); **6.** How helpful do you think an emollient is for your child’s AD? (Significantly, Moderately, Mildly, None); **7.** Do you think emollients specifically for AD are different from normal children’s skin care cream? (Significantly, Moderately, Mildly, None); **8.** How important are an emollient’s ingredients in its effectiveness? (Significantly, Moderately, Mildly, None); **9.** How important do you think it is for an emollient’s ingredients to be non-allergenic? (Significantly, Moderately, Mildly, None)
Rea et al. [38]	Objective Knowledge:	Objective and subjective	Multiple-choice and Likert scale	8 objective and 4 subjective questions	Identical pre- and post-education questionnaires	In clinic for pre-education questionnaire and by telephone or in clinic for post-education questionnaire	Number-right (objective knowledge) and 4-point scale (subjective knowledge)	N/A	**Objective Knowledge Questions:**
	Definition, skincare practices, triggers, bathing, treatment, topical corticosteroids								**1.** Which of the following statements about eczema is true? a) Eczema is always caused by allergies, b) Eczema is a common skin problem in children, c) Eczema can be passed from one person to another by touching, d) Eczema can be cured; **2.** The most important part of eczema skincare is: a) Keeping skin moist by applying cream or lotion, b) Using steroid cream or ointment on the skin, c) Taking anti-itch medicines by mouth, d) Taking antibiotics by mouth; **3.** It is important to avoid these things for children with eczema: a) Scented or fragranced soaps/detergents/lotions, b) Long, hot baths or showers, c) Dry air and sweat, d) Scratchy clothes, e) All of the above; **4.** How should you use your child’s moisturizing cream or lotion? a) Apply a generous layer all over skin, b) Apply a thin layer on red, itchy areas, c) Moisturizing creams and lotions should NOT be used everyday, d) Moisturizing creams and lotions should NOT be used after bathing; **5.** How long should your child’s bath/shower be? a) Less than 10 min, b) 20 min, c) 30 min, d) More than 30 min; **6.** Which of the following is true about your child’s steroid cream or ointment? a) You should apply a generous layer all over the skin, b) You should apply a thin layer only on red, itchy areas, c) Steroid creams and ointments do not have any side effects, d) Steroid creams and ointments should not be used after bathing; **7.** How long after your child’s bath/shower should you apply his/her moisturizing cream or lotion? a) Less than 3 min after the bath/shower, b) 10–20 min after the bath/shower, c) More than 20 min but less than 60 after the bath/shower, d) 1–2 h after the bath/shower, e) Moisturizing creams and lotions should NOT be used after bathing; **8.** When should you call the Children’s Hospital Primary Care Center about your child’s eczema? a) If the itchiness, dryness, redness, flakiness does not improve after 3–5 days, b) If your child develops signs of skin infection including skin oozing/warmth/redness, fever, or not acting like him/herself, c) If you have any concerns about your child’s skin or overall health, d) All of the above
	Subjective Knowledge:								**Subjective Knowledge Questions:**
	Understanding aggravating factors, understanding treatment plan, general eczema understanding, understanding treatment adjustment								**1.** How well do you understand what eczema is? (a) Not at all, b) Somewhat, c) Fairly well, d) Completely); **2.** How well do you understand your child’s treatment plan? (a) Not at all, b) Somewhat, c) Fairly well, d) Completely); **3.** Some things can make a child’s eczema worse, such as scented soap and hot weather. How well do you understand what things make your child’s eczema worse? (a) Not at all, b) Somewhat, c) Fairly well, d) Completely); **4.** Children with eczema often have good skin days and bad skin days. Bad skin days are when the skin is very red and itchy. How well do you understand how to change your child’s eczema treatment when your child is having a bad skin day? (a) Not at all, b) Somewhat, c) Fairly well, d) Completely)
Ryu and Lee [39]	Definition of AD, cause of AD, factors that worsen AD, prevention, treatment and care, nutritional care	Objective	N/A	30	Identical pre- and post-education questionnaires	N/A	Number-right	30 min to complete study questionnaires	N/A
Shi et al. [40]	Individualized treatment plan, benefits and risks of prescribed medication, anatomic location of medication use, duration of treatment, recognizing AD exacerbating factors, adjusting treatment based on disease severity	Subjective	Likert scale	10	Post-education questionnaire only	In clinic	10-point scale	N/A	**1.** Overall, how well do you understand your/your child’s eczema? (0 = I do not understand at all, 10 = I understand completely); **2.** Overall, how well do you understand your/your child’s treatment plan? (0 = I do not understand at all, 10 = I understand completely); **3.** How well do you understand the benefits and risks of the prescribed medication? (0 = I do not understand at all, 10 = I understand completely); **4.** How well do you understand where on the body to apply the medications? (0 = I do not understand at all, 10 = I understand completely); **5.** How well do you understand how long to use the medications? (0 = I do not understand at all, 10 = I understand completely); **6.** Do you understand what factors may make your/your child’s eczema worse? (0 = I do not understand at all, 10 = I understand completely); **7.** Do you understand how to recognize that your/your child’s eczema is well-controlled? (0 = I do not understand at all, 10 = I understand completely); **8.** Do you understand how to adjust your treatment according to the severity of your/your child’s eczema? (0 = I do not understand at all, 10 = I understand completely); **9.** How comfortable are you with the treatment plan for your/your child’s eczema? (0 = I do not understand at all, 10 = I understand completely); **10.** How would you rate your current anxiety level in treating your/your child’s eczema at home? (0 = I do not understand at all, 10 = I understand completely);
Shin et al. [41]	Clinical manifestations, cause/triggering factors, treatment, diagnosis, skin care and environment management (bathing, dietary restriction, breast feeding, exercise, emollient use), common misunderstandings about AD treatment (components and physiologic actions of Chinese herbal therapies, systemic and topical corticosteroid use, water softener and placenta injections)	Objective	N/A	25	Post-education questionnaire only	N/A	Number-right	N/A	N/A
Singer et al. [42]	N/A	Objective	Multiple-choice	16	Identical pre- and post-education questionnaires	On-line	Number-right	N/A	N/A
Son and Lim [4]	Definition, epidemiology, aetiology (including potential trigger factors), symptoms, lesions, assessment (including diagnostic clinical tests for identifying trigger factors), general treatment, skin management (skin hydration, moisturization/emollition and treatment), symptom management (prevention of pruritus and sleep disorders), nutrition management (breastfeeding, weaning food, food allergy and proper nutritional intake for allergy-inducing foods), environment management (management of irritants/trigger factors)	N/A	N/A	N/A	Post-education questionnaires only	Online	N/A	N/A	N/A

## Results

### Selection of sources of evidence

**Fig. 1:**
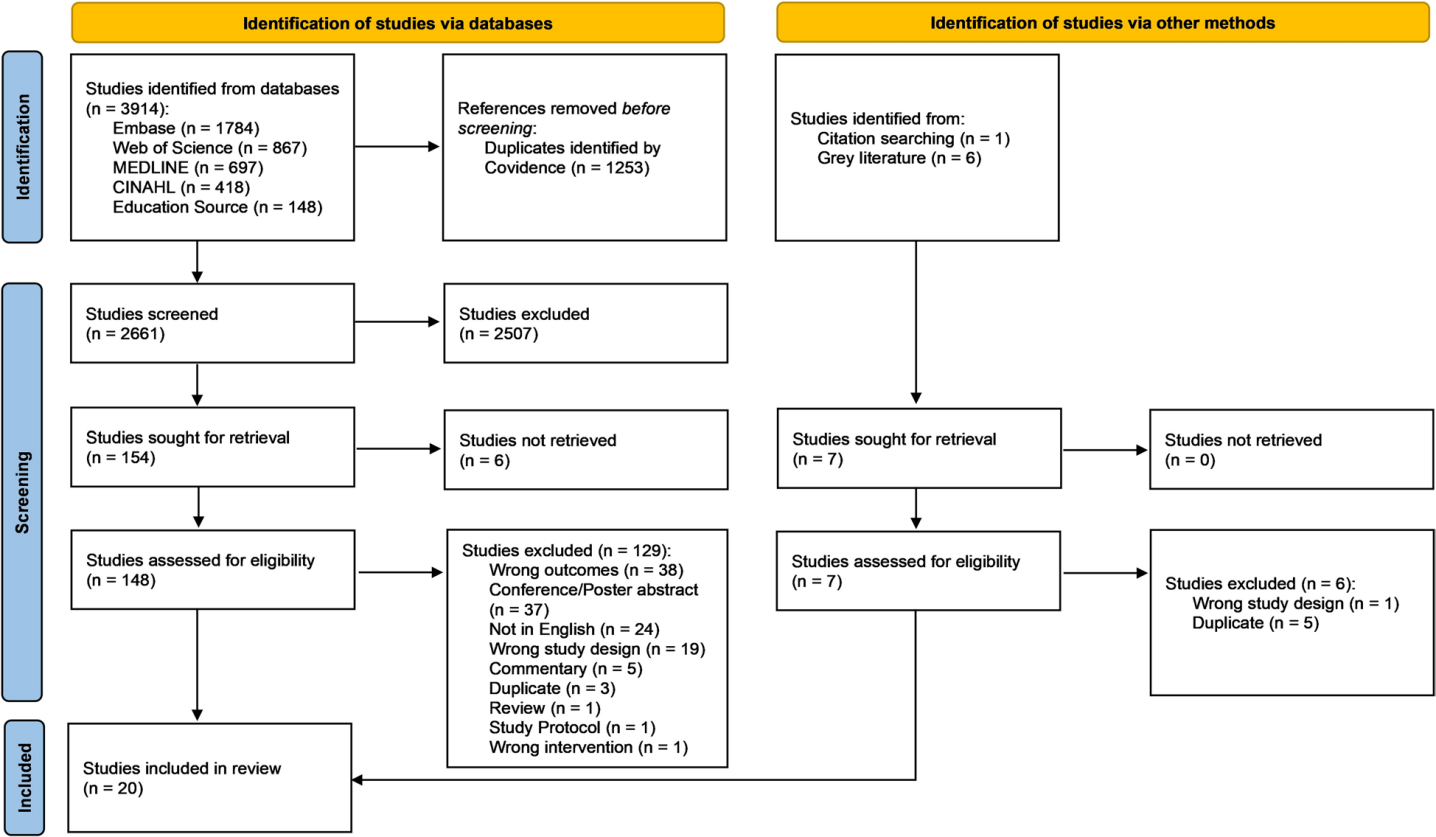
PRISMA-ScR flow diagram depicting numbers of sources of evidence identified, screened, assessed for eligibility, and included in this review

Of 3914 studies identified from databases, 1253 duplicates were removed, and 2661 studies were screened for title and abstract. Of these, 148 full-text publications were assessed for eligibility and 19 studies were included for data extraction. Of 7 studies identified by J.K. and B.W. from citation searching and the grey literature (ClinicalTrials.gov, and ICTRP), 1 was included for data extraction (Fig. 1).

### Characteristics of sources of evidence

A total of 20 studies that assessed patients’ knowledge as an outcome measure of an AD patient education intervention were included in this scoping review [4–7, 13, 20, 31–44]. The characteristics of the sources of evidence included in this scoping review are presented in Table 1. The majority of studies originated in the United States (n = 10) [5, 7, 31, 32, 34, 36, 38, 40, 42, 44], followed by South Korea (n = 4) [4, 39, 41, 43]. The remaining studies were conducted across the United Kingdom (n = 2) [13, 35], France (n = 1) [33], Germany (n = 1) [20], Singapore (n = 1) [6], and China (n = 1) [37]. The articles were published within the last 20 years, with many studies circulating in the last decade. Articles were published in the years of 2023 (n = 2) [31, 32], 2022 (n = 2) [36, 44], 2020 (n = 1) [33], 2018 (n = 5) [6, 7, 37, 38, 42], 2016 (n = 1) [34], 2015 (n = 1) [43], 2014 (n = 4) [4, 20, 39, 40], 2013 (n = 2) [35, 40], 2011 (n = 1) [5], and 2003 (n = 1) [13]. Most studies identified in the search were randomised controlled trials (RCT; n = 11) [5, 7, 20, 32, 34, 37, 38, 40–42]. One publication had a quasi-experimental study design [4], and 8 studies were prospectively designed [6, 13, 31, 33, 35, 39, 43, 44]. The largest study evaluated AD knowledge among 540 participants [37], while the smallest study included 30 participants [42]. The populations represented in the publications of this review ranged from 1 month to 80 years of age. The studies assessed AD caregivers’ knowledge (n = 15) [4, 6, 7, 13, 20, 32, 34–39, 42–44], and AD patients’ knowledge (n = 2) [5, 33]. Three studies assessed both patients’ and caregivers’ knowledge of AD [31, 40, 41]. Thirteen publications evaluated AD patients’/caregivers’ clinical scores as an outcome measure of receiving patient education, in addition to knowledge [4, 5, 7, 13, 20, 32, 33, 36–39, 42, 44]. Disease severity and quality of life was commonly assessed alongside knowledge. Disease severity was evaluated using the EASI (n = 1) [42], SCORAD (n = 6) [20, 32, 33, 36, 37, 39], POEM (n = 4) [4, 5, 38, 44], and Six Area, Six Sign Atopic Dermatitis Severity Score (SASSAD; n = 1) [13]. Quality of life was measured using the Children’s Dermatology Life Quality Index (CDLQI; n = 4) [7, 37–39], and Infants’ Dermatitis Quality of Life Index (IDQOL; n = 4) [4, 7, 37, 38].

### Content of AD knowledge assessment tools

Sixteen studies detailed the topics and content comprising knowledge assessment tools of AD patient education interventions [4–6, 20, 31, 33–41, 43, 44]. Of these, 11 publications determined AD patients’ understanding of the definition, cause, and clinical manifestations of AD [4–6, 31, 33, 35, 38, 39, 41, 43, 44]; and 10 studies evaluated patients’ understanding of environmental triggers of AD [4–6, 31, 33, 35, 38–41]. Cheong et al., included the definition of an eczema flare as a topic of their knowledge assessment tool, and Son et al., assessed patients’ knowledge of diagnostic clinical tests for identifying trigger factors of AD [4, 6].

Questions on pharmacological and non-pharmacological treatments for AD were included in the knowledge assessments of 13 studies [4–6, 20, 31, 34–41]. Items related to the use of topical corticosteroids and emollients were commonly included in AD knowledge assessment tools. One study evaluated patients’ knowledge of the anatomic location of medication use and the duration of treatment, in addition to the benefits and risks of using prescribed medications [40]. Patients’ knowledge of preventative measures for AD was assessed in 6 knowledge assessment tools and included questions on adjusting treatment based on disease severity, as well as mechanisms for preventing pruritus and sleep disorders [4, 34, 36, 38–40]. A single study included questions in their knowledge assessment tool related to assessing patients’ understanding of common misconceptions about AD treatment. These questions were related to common misunderstandings about systemic and topical corticosteroid use (i.e., corticosteroid phobia), and the use of alternative medicines (i.e., the components and physiologic actions of Chinese herbal therapies, water softener and placenta injections) [41].

Skin care practices and management for AD, including moisturizer vehicles and use were assessed in 7 knowledge assessment tools [4–6, 33, 34, 38, 41]. Three instruments included questions that evaluated patients’ knowledge for recognizing skin lesions [4, 33, 36]. Bathing and washing techniques were also appraised in four knowledge assessments [5, 36, 38, 41], with Joseph et al., evaluating patients’ knowledge of wet wrap therapy and bleach baths for AD.

Five studies included the topic of diet in their AD knowledge assessment tools [4, 20, 31, 39, 41]. Questions were related to assessing patients’ knowledge of dietary restrictions, food allergy and proper nutritional intake for allergy-inducing foods, as well as breastfeeding and weaning food for mothers and/or infants with AD. One study included questions regarding the role of exercise in AD in their knowledge assessment tool [41]. Additionally, Andrade et al., and Breuer et al., incorporated the topic of psychological impacts of AD in their knowledge assessments, and evaluated patients’ understanding of stress reduction and coping mechanisms [20, 31].

Studies employed various methods to decide upon the topics to include in their knowledge assessment tools. Questionnaire items of two knowledge assessment tools were drawn from the contents of the patient education materials [5, 31]. The content of one knowledge assessment tool was developed by expert dermatologists in the field of AD, with questions simplified to be understood at the middle school reading level [31]. To determine the content of instructional items and study questionnaires, Rea et al. solicited feedback from families and primary care providers to assess which aspects they thought were most important and helpful to include [38]. In contrast, content for the knowledge assessment tools of 3 publications were taken from previous studies, research and/or textbook entries [36, 39, 41] (Table 2).

### Types of knowledge assessed in AD patient education interventions

Eight studies assessed AD patients’ objective knowledge [6, 20, 31, 33, 38, 39, 41, 42] and 9 publications measured patients’ subjective knowledge [7, 34–38, 40, 43, 44]. Rea et al. [38] evaluated patients’ objective knowledge through a series of fact-based questions, and patients’ subjective knowledge by relying on caregivers’ assessment of their own eczema knowledge and understanding (Table 2).

### Types of questions in AD knowledge assessment tools

Question structure and formats of objective and subjective knowledge assessments varied in the literature. Of the articles which assessed patients’/caregivers’ objective knowledge, dichotomous (i.e., true/false, right/wrong; n = 4) [6, 20, 31, 33] and multiple-choice (n = 3) [33, 38, 42] formats were primarily used to structure questions. One study used both multiple-choice and true/false question formats in their objective knowledge assessment tool [33]. Likert scales were commonly used to format questions of AD subjective knowledge assessment tools (n = 8) [7, 34, 36–38, 40, 43, 44]. Additionally, a single study assessed patients’/caregivers’ subjective knowledge and understanding of an Eczema Education Program intervention through focus group interviews [35] (Table 2).

### Number of questions comprising AD knowledge assessment tools

Twelve studies included a varying number of questions in their knowledge assessment tools. The lowest number of questions included in a knowledge assessment was 1 question [43], and the highest number of questions comprising an AD knowledge assessment tool was 49 questions [20]. Researchers assessing patients’/caregivers’ objective knowledge included a greater number of questions (ranging from 8–49 questions) in their knowledge assessments than those which assessed patients’/caregivers’ subjective knowledge (ranging from 1–10 questions). Notably, Rea et al. [38] included twice as many objective knowledge questions than subjective knowledge questions in their knowledge assessment (Table 2).

### Characteristics of pre- and post-education AD knowledge assessment tools

Thirteen studies had identical pre- and post-education knowledge assessments [5–7, 13, 20, 31–33, 36–39, 42]. Cheong et al. reversed the order of questions in their post-education knowledge assessment completed by patients/caregivers 4 weeks after education [6]. Six publications had a post-education knowledge assessment only [4, 35, 40, 41, 43, 44], and one article had a post-education knowledge questionnaire identical to 1 month and 3 month follow-up questionnaires that were administered afterwards [34] (Table 2).

### Delivery mode of AD knowledge assessment tools

Three studies delivered their AD knowledge assessments to participants online [4, 31, 42], 4 studies delivered it in the clinical setting [13, 35, 36, 40], and 2 studies administered knowledge questions to participants by telephone [43, 44]. Cork et al. distributed their knowledge assessment to patients/caregivers in clinic on paper, which had been validated and adapted in the same clinic over the preceding six months [13]. Additionally, four studies delivered their pre-education/baseline knowledge assessment in clinic, and verbally administered their post-education knowledge assessment by telephone [6, 7, 34, 38]. Rea et al. [38] delivered their post-education knowledge assessment in clinic and/or by telephone to patients/caregivers (Table 2).

### Scoring methods for AD knowledge assessment tools

Six knowledge assessments evaluating patients’/caregivers’ objective knowledge had questions graded by the number-right scoring method [20, 31, 38, 39, 41, 42]. Two studies converted the number of questions answered correctly into a score out of 100 points for their assessments [38, 39]. Cheong et al. [6] employed the negative scoring method for their knowledge assessment tool, where each correct response was awarded 1 point, an incorrect response was graded negative 1 point, and a “Not sure” answer was awarded no points. Eight studies assessing patients’/caregivers’ subjective knowledge scored the average of responses to questions on a 4-point (n = 2) [37, 38], 5-point (n = 5) [7, 34, 36, 43, 44], or 10-point (n = 1) Likert-type scale [40] (Table 2).

#### Time to complete AD knowledge assessment tools

A single study by Ryu and Lee [39] indicated that it took approximately 30 min to complete their knowledge assessment. Although, the 30-min completion time accounted for other study questionnaires completed by participants in addition to the knowledge assessment, including a 9-item Parental Efficacy test, 21-item Parent Compliance scale, and 14-item Atopic Dermatitis Impact Scale [39] (Table 2).

## Discussion

### Summary of main findings

This scoping review aimed to identify and characterize the literature on knowledge assessment tools for AD patient education interventions. Twenty studies were included in this review, which assessed knowledge as an outcome measure of an AD patient education intervention. Methods for evaluating AD patients’/caregivers’ objective knowledge were determined to be consistent across studies with minimal variability. Likewise, studies which assessed patients’/caregivers’ subjective/self-perceived knowledge used similar methods for formatting and structuring their knowledge assessment tools. Objective AD knowledge assessment tools were commonly structured to include questions in multiple-choice and/or true/false formats, with items graded by the number-right scoring method. Objective AD knowledge assessment tools had more questions than subjective AD knowledge assessments. Likert-type scales were primarily used to score and format questions in subjective AD knowledge assessment tools. Common aspects of AD and its management were evaluated in both objective and subjective knowledge assessment tools, with studies emphasizing assessment of patients’/caregivers’ knowledge of the disease, pharmacological and non-pharmacological treatments, as well as environmental triggers. Additionally, subjective and objective knowledge assessments in pre-test—post-test study designs were identical in content, format, and structure, and delivered through similar modes in clinic, online, and/or by telephone.

### Future directions

Despite studies using comparable methods for assessing AD patients’/caregivers’ objective knowledge, and similar approaches across studies which measured their subjective knowledge, an optimal and/or unified conceptual method for evaluating AD knowledge in patient education interventions has not been presented in the literature to date. Subjective and objective knowledge assessment tools can provide indications for different learning outcomes and achievements of patients after receiving education [45, 46]. Subjective/‘Divergent’ knowledge assessments are associated with qualitative changes in an individual’s perception of their knowledge, learning, and/or growth, whereas objective/‘convergent’ knowledge assessment tools can be used for assessing quantitative changes in one’s acquired and/or factual knowledge [45, 46]. A discrepancy also exists between one’s actual knowledge and their self-assessment of that knowledge, as individuals with a low level of actual knowledge may have a high perception of their knowledge or cognitive abilities, and vice-versa [47, 48]. Given the absence of a preference for the use of subjective or objective AD knowledge assessment tools within the literature and the differences in their purported use, researchers should develop knowledge assessment tools in accordance with the specific learning outcomes of AD patient education interventions [45, 49].

Designing knowledge assessment tools for patient education interventions is a nuanced, complex, and intricate process which requires consideration for various factors, such as individual differences in learning and/or learning styles (ex. visual, verbal, physical, social, aural, logical, and solitary), literacy, education, age, and gender [50]. These factors were not adequately detailed and/or accounted for in the studies included in this review, with only one study constructing their AD knowledge assessment tool to be understood at the middle-school reading level [31]. Individual differences in learning have shown to affect one’s performance on knowledge tests, and a failure to account for this in the design process could potentially result in testing bias [51, 52]. Gender bias is an example of such bias, which may arise from using negative scoring methods [6, 53, 54]. Future research should construct AD knowledge assessment tools which are diverse and comprehensive to meet the population’s needs. Pilot testing AD knowledge assessment tools can identify knowledge gaps in patients and can discover potential problems or deficiencies in the measure prior to its study implementation [50].

Designing knowledge assessment tools also requires consideration for the selection of an appropriate assessment method. The field of medical education widely employs multiple-choice question formats for assessing individuals’ objective knowledge [55]. Well-constructed multiple-choice knowledge assessments can be used to assess higher-order cognitive processes of patients, such as the application of patients’ knowledge to the context of self-managing their disease, as opposed to evaluating their recall of isolated facts [55]. Multiple-choice knowledge assessments are efficient for testing a wide breadth of content and may also enhance long-term retention of material learned during patient education interventions for subjects [56]. However, some variations of multiple-choice questions such as true/false questions may not be suitable for assessing knowledge in AD patient education [57]. True/false knowledge assessment tools may increase the likelihood of participants guessing the correct answer and can discourage subjects from learning around the questionnaire items compared to traditional multiple-choice knowledge assessments (i.e., question stem and a set of options in which one is only correct) [57, 58].

A single method of assessment may not be sufficient for evaluating all the knowledge, skills, and attitudes of participants after AD patient education [55]. For instance, evaluating AD patients’ objective knowledge after education through multiple-choice assessments can provide insight into whether patients understand their disease and the skills and lifestyle changes that they need to implement to better manage their disease [59–61]. Whereas subjective knowledge assessments evaluated by Likert scales may provide a greater indication of patients’ confidence and beliefs in their abilities to act on their knowledge and change their behaviours and attitudes accordingly to improve their health outcomes [61, 62]. In either case, knowledge assessments would need to be supplemented with the use of objective clinical outcome measures to determine the impact of AD patients’ subjective and/or objective knowledge on their health outcomes and ability to effectively manage and control their disease. Future research may consider using a combination of assessment techniques to evaluate the knowledge of patients after receiving formal patient education.

The delivery mode and questions comprising pre-post test knowledge assessments can influence the quality and reliability of data obtained [63]. Four studies altered the delivery mode for their post-education knowledge assessment in relation to the pre-education assessment [6, 7, 32, 36]. Variations in the delivery mode of AD knowledge assessment tools can introduce survey error or bias (i.e., mode effect), and alter the types of responses provided by respondents to questionnaires [63]. A similar mode of delivery should be retained for delivering knowledge assessment tools pre- and post-education [63]. Additionally, while the literature has shown that repeated testing after education can aid in long-term knowledge retention (i.e., test-enhanced learning) compared to repeated instruction or no testing, all studies included in the review presented the same knowledge questions at baseline as they did at post-education/follow-up, which limits the ability to distinguish between knowledge acquisition and knowledge recall [40, 64–66]. Further research should consider delivering alternate forms of AD knowledge assessment tools that measure the same construct equally well over different time points to evaluate knowledge retention [67].

Developing high-quality knowledge outcome measures for AD patient education is dependent upon the language, ease of comprehension, and length and number of questions [68]. Few studies specified the time required by patients to complete AD knowledge assessments after receiving patient education, and the length and number of questions comprising subjective and objective knowledge assessment tools varied in the literature. The length and number of questionnaire items and time to complete knowledge assessment tools can significantly affect the response rates, and the quality and reliability of data obtained for assessing the effectiveness of AD patient education interventions [68]. Within the confines of a busy clinical setting, there is often a limited amount of time which dermatologists and/or other healthcare providers have for delivering AD patient education interventions and assessing their respective outcomes [69, 70]. AD patients may also exhibit ‘information overload’ by acquiring too much information at a single timepoint from the information received during the patient education intervention in addition to the information received regarding the reason(s) for their medical visit [69]. As AD patients become over-burdened with information, they may become fatigued and concomitant response errors may occur, especially in the case of having to complete lengthy questionnaires following their medical visit and/or receiving a patient education intervention [69]. To ensure that knowledge outcome measures for AD patient education are feasible for delivery and completion in the clinical setting, questions should be simplified by using shorter words and sentences written in plain language, to aid in the knowledge recall of patients [69]. Similar to developed and validated knowledge assessment tools for other chronic diseases, such as asthma [71], diabetes [72], and Crohn’s disease [73], AD knowledge assessment tools should ideally be composed of 25–30 questions and administered within 30 min to reduce the likelihood that participants speed up or “satisfice” (i.e., the tendency for respondents to take short-cuts in answering questions by producing satisfactory rather than optimal responses) through the questions, and ensure that they remain interested and attentive while responding to the questionnaire [68]. The length and number of questions comprising knowledge assessment tools for other chronic diseases have varied in the literature, similarly to AD. The length and number of questions to include in knowledge assessment tools should ultimately be based upon best research practices for delivering assessments within the settings in which they will be delivered.

The limited studies supporting the use of knowledge assessment tools as outcome measures of AD patient education interventions and the lack of validated measures, emphasizes the need for further research in this area. Two studies in this review validated their knowledge questionnaires prior to study implementation [5, 13]. However, the studies validation methods and/or content of their knowledge assessment tools were not adequately detailed to critically appraise their quality. Additionally, Sun et al. developed and validated a subjective knowledge questionnaire assessing parents’/caregivers’ knowledge of infant AD skincare, attitudes and practices [74]. However, questionnaires such as the latter may only assess a specific aspect of the disease, thereby limiting the ability to repurpose the knowledge assessment tool for other patient education interventions that may consist of more content. Content of knowledge assessment tools should be in alignment with the AD patient education intervention it intends to measure, otherwise it risks a lack of continuity which could potentially raise concerns about the reliability and validity of the measurement and study [24, 49]. A greater amount of time may also be required by patients to complete knowledge assessments which are not in alignment with the content of the education intervention they received [49]. Additionally, knowledge assessments are valid for a particular group of people within a particular context, and a study’s validated AD knowledge assessment tool may not be suitable for use for a different patient education intervention or for a different population [75]. This warrants further developmental and validation studies on AD knowledge assessment tools for patient education interventions.

## Strengths and limitations

The scoping review encompassed a broad search strategy with relevant databases from a variety of disciplines, as well as sources of evidence from the grey literature and clinical trial registries. This established best efforts to capture all relevant articles for the review. Additionally, citation searching in accordance with the snowball search method was employed to minimize the exclusion of articles. A limitation of this review was the exclusion of articles not written in the English language, which may have resulted in the exclusion of relevant articles. Since the review included articles that assessed knowledge as an outcome measure of an AD patient education intervention, publications which evaluated AD patients’ knowledge for other purposes were not accounted for. Additionally, the included sources of evidence primarily originated in Western parts of the world, thereby limiting the generalizability of this review to other contexts.

## Conclusion

This scoping review synthesized the existing body of literature on AD knowledge assessment tools for patient education interventions. The importance of AD patient education for improving patients’/caregivers’ knowledge, self-efficacy, and self-management practices underscores the need for outcome measures which can directly assess their impact. Knowledge assessment tools are valuable for directly assessing the effectiveness of AD patient education interventions for patients/caregivers. The development of AD knowledge assessment tools requires methodological rigor and careful consideration for various factors throughout its design process and implementation. Well constructed knowledge assessment tools can have important implications for identifying AD patients’/caregivers’ educational needs, knowledge deficiencies, and/or areas of improved knowledge and understanding as a result of receiving education. The limited availability of validated knowledge outcome measures in AD literature presents challenges towards assessing the true effects of patient education interventions on improving patients’/caregivers’ knowledge. Further studies guided by pedagogical practices and pragmatism are needed for developing and validating high-quality AD knowledge assessment tools for patient education interventions.

## Data Availability

No datasets were generated or analysed during the current study.

## Abbreviations

AD: Atopic Dermatitis
ICTRP: International Clinical Trials Registry Platform
PROMs: Patient-reported Outcome Measures
HOME: Harmonizing Outcome Measures for Eczema
EASI: Eczema Area and Severity Index
SCORAD: SCORing Atopic Dermatitis
POEM: Patient Oriented Eczema Measure
DLQI: Dermatology Life Quality Index
ADCT: Atopic Dermatitis Control Tool
RECAP: Recap of Atopic Dermatitis
PRISMA-ScR: PRISMA Extension for Scoping Reviews
RCT: Randomised Controlled Trials
SASSAD: Six Area, Six Sign Atopic Dermatitis Severity Score
CDLQI: Children’s Dermatology Life Quality Index
IDQOL: Infants’ Dermatitis Quality of Life Index
